# Editorial: Drug metabolism-induced organ toxicity

**DOI:** 10.3389/fphar.2024.1362229

**Published:** 2024-01-25

**Authors:** Adnan A. Kadi, Yang Li, Abhinav Parashar, Mohamed W. Attwa

**Affiliations:** ^1^ College of Pharmacy, King Saud University, Riyadh, Saudi Arabia; ^2^ Division of Pharmaceutics and Pharmacology, College of Pharmacy, The Ohio State University, Columbus, OH, United States; ^3^ Department of Biological Sciences, P. D. Patel Institute of Applied Sciences, Charotar University of Science and Technology, Changa, Gujarat, India

**Keywords:** drug metabolism, organ toxicity, adverse drug reactions, reactive intermediates, bioactivation

Drug Metabolism refers to the process of detoxifying xenobiotics and endogenous molecules by converting them into more hydrophilic substances, which enables their elimination from the body. Drug metabolism is an integral part of the pharmacokinetic parameters of a given drug entering the living organism and, together with absorption, distribution, and elimination, constitute the four main parameters of pharmacokinetics, known as ADME. In the pharmaceutical industry, studying the metabolism of a given molecule is a crucial phase in the drug discovery process and often determines the success of a medicine in obtaining approval and reaching the market. Organ toxicity mediated by drug metabolism represents a challenge in the drug discovery process and is considered a research area that would gain more importance from further work in this important field. The occurrence of drug-induced liver injury is the most common among metabolism-mediated organ toxicities and is usually the principal reason for post-marketing withdrawals. Given the remarkable advances over the past 2 decades in analytical technologies (notably liquid chromatography-tandem mass spectrometry, LC-MS/MS) for the detection, identification, and quantitative analysis of drugs and metabolites in biological fluids, it is important to characterize the metabolic profile of a new chemical entity in animals and human subjects.

The majority of medications are xenobiotics, meaning they are chemical molecules that are not naturally synthesized by the body. Xenobiotics undergo a range of physiological mechanisms to detoxify them, thereby decreasing their toxicity and facilitating their elimination from the body (Zhang et al.). These processes enable the chemical alteration of pharmaceuticals into their metabolites and are referred to as drug metabolism or metabolic biotransformation (Karolyi et al.). These metabolites are the result of medication metabolism and can be classified as active, inactive, or hazardous. Active metabolites are biologically active molecules that have therapeutic effects, while inactive metabolites are biologically inert compounds that have neither therapeutic nor harmful effects. Toxic metabolites are biologically active substances that resemble active metabolites and can have a range of detrimental effects (Wang et al.). Reactive metabolites are well acknowledged to have a crucial role in the occurrence of idiosyncratic adverse drug reactions (IDR) (Wang et al.). Reactive metabolites participate in numerous mutagenic/carcinogenic occurrences through their interaction with DNA.

The process of selecting drugs for clinical trials typically begins with a vast array of varied hit/lead compounds, which are carefully examined and refined over a period of several years. At the conclusion of this process, a small number of structures that have been optimized for effectiveness, ADME characteristics, and a lack of immediate toxicity should have been identified. It is important to note that when selecting a candidate drug, it is acceptable to presume that the structural characteristics of the candidate drug that are most important for activation of reactive species have already been established. Foundational understanding may have been derived from a xenobiotic or any other molecule that is reported to have undergone an enzymatic transformation into confirmed or hypothesized reactive intermediates. Although the recognition of structural alarms may appear challenging for any project team, it should nevertheless be given careful consideration. The development of most reactive species can, in theory, be anticipated by drawing on the existing knowledge of documented cases and using theoretical analysis of bond building and breaking activities, whether through enzymes or other means, such as direct electron-transfer processes. Assessing the danger against benefit involves evaluating the likelihood of an activation occurring to a substantial degree in humans. This factor, along with the dosage/exposure, will determine the amount of substance accumulated in the body, which is currently estimated to a considerable extent. Collectively, these factors will define the severity of the presence of a substance of concern.

Drug designers should benefit from all published data that is organized in a way that is readily searchable, starting with a query structure using different *in silico* software. An increased number of structural alerts with more details is used to attain a good decision. There is a link between the chosen structural alerts and cited drugs, whose predicted activation mechanisms depend on the knowledge base. Drug designers have strong incentives to exert significant efforts in order to prevent the production of reactive metabolites in test compounds.

